# Cross-country adaptation and feasibility of an evidence-based resistance training intervention in the school setting

**DOI:** 10.3389/fspor.2024.1415469

**Published:** 2024-08-08

**Authors:** Caroline R. Hartman, David R. Lubans, Lars B. Christiansen

**Affiliations:** ^1^ Department of Sports Science and Clinical Biomechanics, University of Southern Denmark, Odense, Denmark; ^2^ Centre for Active Living and Learning, College of Human and Social Futures, University of Newcastle, Callaghan, NSW, Australia; ^3^ Hunter Medical Research Institute, New Lambton Heights, NSW, Australia; ^4^ Faculty of Sport and Health Sciences, University of Jyväskylä, Jyväskylä, Finland

**Keywords:** adolescent, muscular fitness, physical activity, physical education, skill competency

## Abstract

**Background:**

Physical activity that strengthens muscles and bones at least three times per week are recommended, but few adolescents meet this target. The aim of our study was to adapt and evaluate the feasibility and preliminary efficacy of the Resistance Training for Teens (RT4T) program in Danish lower secondary schools.

**Methods:**

Developed and evaluated in Australia, the aim of RT4T is to provide adolescents with competence, confidence, knowledge, and motivation to participate in resistance training. Translation and adaptation were based on the PRACTIS-guide and involved workshops with physical education teachers. Three 7th-grade classes and three 9th-grade classes were recruited for the feasibility study and followed the program over eight weeks. Participants completed a push-up test, a standing long jump test, and a beep-test before and after the intervention. In addition, they completed a survey about their self-efficacy, motivation, and resistance training competency. Four qualitative interviews were performed with participants and their physical education teachers.

**Results:**

The participating teachers were motivated for the program, but they had difficulties providing students with appropriate feedback. Students were motivated by the equipment, games, and their improvements in fitness, but motivation declined during the program. A total of 57 students completed the physical tests and answered the survey. Resistance training self-efficacy improved significantly, while most other measures improved over time, without reaching significance.

**Conclusion:**

Overall, the RT4T was acceptable and feasible in lower secondary schools in Denmark, but adjustments need to be made to increase the reach and efficacy of the program.

## 1 Introduction

The World Health Organization (WHO) recommends children and adolescents participate in an average of 60 min of moderate-to-vigorous physical activity every day, with three sessions of muscle and bone strengthening activity every week (1). Although the benefits of aerobic activity for young people are well established (2), there is an emerging evidence base highlighting the importance of muscle-strengthening activities for children and youth (3–5). Recent reviews have demonstrated that young people's muscular fitness is associated with improved fundamental movement skill competency, mental health, cognitive performance, and cardiometabolic health (6–10). However, few adolescents meet the muscle strengthening guidelines and reap the extensive benefits of this form of physical activity (11, 12).

Schools have previously been pointed out as key settings for physical activity promotion, and this is also the case, when it comes to resistance training (13). In many countries “physical fitness and training” is included in physical education curricula, but it is not known to what extent teachers implement resistance training in their classes. A review of school-based studies has found that resistance training among youth can yield positive results (9). Various methods, such as calisthenics, circuit training and suspension training, have led to significant improvements in strength and physical competencies (14, 15). Moreover, several studies have found that school-based resistance training can improve students' perceived fitness, self-efficacy, and motor competence (8, 16). In conclusion, these studies highlight the value of incorporating resistance training into school physical education programs.

During the last decade, a research team at the University of Newcastle in Australia has developed and evaluated a range of school-based resistance training programs (17–19). This work has resulted in an evidence-based program called *Resistance Training for Teens* (RT4T), which is being disseminated in Australia and beyond. The program was found to be effective in a cluster randomized controlled trial involving 16 schools and was then scaled-up across New South Wales, Australia (20, 21). In the scale-up study, 249 schools and around 10,000 adolescents participated in RT4T (20).

The aim of the RT4T program is to provide adolescents aged 12–16 years with competence, confidence, knowledge, and motivation to participate in foundational resistance training. The program consists primarily of a 90-minute session of fun and engaging muscle strengthening activities delivered by teachers. The sessions are supported by circuit cards, equipment, a purpose-built smartphone application (app), and training materials. Furthermore, the sessions have been supplemented by student seminars, lunch time fitness sessions and incorporated broader health messages (17, 20, 21). Finally, teachers are provided with training to deliver the sessions using the SAAFE principles (Supportive, Active, Autonomous, Fair and Enjoyable). These principles are designed to promote a psychologically supportive environment, foster a mastery climate, and enhance young people's autonomous motivation (22). The RT4T program follows the recommendations for resistance training for youth: include warm-up and cool-down, supervised resistance training by a trained teacher, prioritize the technical aspect, provide adolescent-size equipment or equipment where it is possible to prioritize a good technique (5).

In Denmark, Physical Education (PE) is part of the curriculum for 135 min per week in lower secondary school (7th to 9th grade). During PE, the students should develop physical, athletic, social, and personal competences and grow motivation for lifelong physical activity (23). Knowledge and skills related to resistance training is part of the national learning objectives belonging to the content area *physical exercise,* where the focus is on various forms of exercise, creating warm-ups and training programs for muscles, cardiovascular system, flexibility, strength, and endurance. Teacher have no standardized curriculum and prioritization and quality of resistance training education varies between teachers as many have limited content knowledge, as seen in other countries (13). The RT4T could provide an evidence-based approach for promoting resistance training in Danish schools. Therefore, the main aim of our study was to adapt the Australian RT4T program and assess the feasibility of the program in Danish secondary schools. Our secondary aim was to examine the preliminary efficacy of the adapted program.

## 2 Methods

Our study was designed using the CONSORT checklist for pilot trials (24) (Supplementary File) and the guidelines for reporting feasibility trials (25). Our method section will include information on the translation and adaptation of the original Australian trial as well as the information on the feasibility trial in Denmark.

### 2.1 Trial design

Our feasibility trial was a mixed methods 8-week uncontrolled trial. We collected and analyzed quantitative and qualitative data to address the aims of the study. The quantitative data consisted of a survey and physical tests prior to and after the 8-week intervention. The qualitative data consist of interviews with the teachers after the intervention.

### 2.2 Participants

Schools were recruited through invitations send to the school managers of local schools in the Copenhagen area and followed up by personal phone calls from April to June 2022. Additionally, an open invitation was shared on LinkedIn, newsletters, and website. Eligible teachers were those teaching PE to students in the upper grades (7th-9th grade) willing to allocate the necessary time to deliver RT4T during PE classes. Furthermore, the teachers should be able to participate in a training and adaptation workshop. Since this was a pilot study, we did not conduct a sample size calculation. We aimed to recruit classes from different grades (7th to 9th) and from at least two schools to ensure diversity and reasonable sample size to inform us about the feasibility of delivering the RT4T program in Danish schools. Two schools responded to the invitations and participated in the project from August to October 2022 after conducting the 8-week program. One school participated with three 7th-grade classes and the other with three 9th-grade classes. All students with parental consent were eligible in the study. In Denmark, lower secondary schools consist of 7th to 9th grades, and the students were aged 12–16 years.

### 2.3 Intervention

#### 2.3.1 Translation and adaptation

The Australian RT4T program was translated and adapted to the Danish school context using the PRACTIS-guide (26). The guide consists of four steps: (1) understand the implementation setting, (2) engage key stakeholders, (3) identify barriers for implementation and (4) address barriers for implementation. As part of the first step agreement and collaboration were established with the research team at the University of Newcastle. They provided original material, resources, and guidance on the adaptation and implementation to the Danish context. Here the experiences with the planning and implementation processes from the original studies were identified.

PE teachers were considered to be the key stakeholders in the planning phase, and students were involved after the program in the formative evaluation. One teacher from each school participated in a 2-hour training and implementation workshop where they were presented for the WHO's recommendations for physical activity and muscle strengthening, the evidence base for RT, and the RT4T program. Participating teachers received a protocol after the workshop. A protocol consisting of instructions about RT4T and procedures for the planned tests and surveys prior to and after the intervention. As part of the workshop, teachers were involved in the implementation process and assisted in identifying the implementation facilitators and barriers. The teachers were asked to write positive and negative thoughts down on post-it notes and put them on a poster. Afterwards the notes were discussed in relation to possible solutions and successful implementation. One important facilitator in program, was the flexible structure and possibilities to choose different activities. The main adaptations of the intervention were made in agreement with the Australian research group and are listed below. The description of the intervention are elaborated in a later section:

• Due to organization in period between holidays the program was delivered in 8 weeks instead of 10 weeks.
• Lunch time activity was implemented with different results in the Australian program and to reduce load on teacher it was removed from the Danish version.
• To reduce cost and complexity suspension straps or agility ladder was removed from the equipment package.

The original study included motivational healthy messages (e.g., avoid sugary drinks), which were left out due to the specific focus on resistance-training related outcomes.

Based on their review of the program, the teachers affirmed that it did not contain potentially harmful elements and that the activities align with what students are normally presented with in their classes. Additionally, the activities are primarily based on body weight and resistance bands, and the focus is specifically on a supportive social environment. However, we did not investigate potential harmful consequences, and future studies should integrate this perspective.

#### 2.3.2 Materials and resources

The recruited schools were provided with access to the smartphone app and an equipment package with five sets of boxing gloves and pads, eight Gymsticks™ with resistance bands, four training bands in two different levels, and three sets of circuit cards. Moreover, the schools supplemented with own materials and provided an open area (sports or gym hall) to run the RT4T program.

#### 2.3.3 Intervention delivery

At the start of the intervention, the teachers conducted an interactive seminar introducing students to the 8-week program. The seminar included presentation of the national physical activity recommendations and the benefits of resistance training as well as interactive parts where students were invited to reflect and share thoughts on physical activity and resistance training. In the current trial, the intervention was limited to one 45–60-min RT4T session/week delivered during PE classes. The teachers from the implementation workshops were assisted by 1–2 additional PE teachers during the sessions, who received the manuals and circuit cards. The teachers were strongly encouraged to follow the SAAFE principles when delivering sessions to promote motivation for physical activity. The SAAFE principles were developed using self-determination theory and focus on building a supportive environment with an emphasis on student enjoyment, autonomy, and differentiation (22). Each session included the following structure: Warm-up, GymFit, and Cool Down and by the choice of teachers and students either HIRT, GameFit, BoxFit, CardioFit, and/or CoreFit (Table 1). Warm-ups consisted of four kinds of dynamic stretching and a short movement bases game. The aim of GymFit was to develop students’ resistance training skill competency. The teachers were recommended to prepare at least 6–8 exercises covering horizontal push, vertical pull, horizontal pull, lower body bilateral, lower unilateral, and trunk stability for full body training. The progression, sets, and reps were based on the existing consensus and guidelines (5, 10). HIRT (High Intensity Resistance Training) were done in pairs and focused on improving students' muscular and cardiorespiratory fitness. This was followed by a selective part with multiple activities and allowed for students' choice, which included a range of different activities e.g., games, yoga, boxing, and Pilates. Finally, the sessions were ended by a cool down with stretching and evaluation of the training.

**Table 1:** The structure of the RT4T sessions.

Activity	Purpose	Explanation	Duration
Warm-up	Safety	General warm-up involving movement-based games and dynamic stretching	3–5 min
GymFit	Develop resistance training movement skills	Workout consisting of for example Gymsticks™/bands and body weight exercises with moderate intensity.	20–30 min
HIRT	Improve muscular and cardiorespiratory fitness	Short, high-intensity workout	8–26 min
BoxFit, CardioFit, CoreFit or GameFit	Enjoyment and students’ choice	Session of e.g., boxing, circuit training, Pilates or modified game with fitness focus.	20–30 min
Cool Down	Reflection and restitution	Static stretching and light activity including evaluations of the training.	5 min

The original structure of RT4T can be accessed here: http://links.lww.com/MSS/B19 (21).

### 2.4 Data collection

The data collection of the feasibility trial consists of three main parts related to the overall aims: student survey, physical fitness test and interview with teachers.

#### 2.4.1 Translation and adaptation

The original student survey and physical test protocols were made available by the research team from University of Newcastle, Australia. They also provided guidance and advice on how the program could be adapted to the Danish context. The current trial focused on resistance training alone and therefore parts related to nutrition, sleep, and health behavior were excluded from the study. The questionnaire went through a translation, backtranslation and comparison process from English to Danish informed by published guidelines (27). The reliability of translated scales consisting of several items was assessed using Cronbach's alpha at baseline. The original physical test protocol was followed except a replacement of the step test to beep-test for aerobic performance.

#### 2.4.2 Student survey

Before and after the 8-week trial, the students answered an individual online survey related to these areas: physical activity behavior, motivation and self-efficacy related to physical activity and resistance training, general well-being, and self-efficacy. To evaluate if students met the youth physical activity guidelines, a validated single-item physical activity questionnaire was used (28). The five-item International Fitness Scale was used to assess students’ perceptions of their strength, speed, flexibility, cardiorespiratory, and general fitness (29). Students' resistance training self-efficacy was assessed using the four-item Resistance Training Self-Efficacy Scale (30). The scale demonstrated good internal consistency with a Cronbach's alpha of 0.81. Selected items from the BREQ-2 were used to evaluate the students' motivations for regular exercise. The two subscales and a total of eight items were used to assess intrinsic and identified regulation (31). The subscales had Cronbach's alpha at 0.86. and 0.84, respectively. Finally, Diener's Psychological Flourishing Scale of eight items was used to evaluate students' general well-being (32). This translated scale showed also good internal consistency with a Cronbach's alpha at 0.83. After the trial, students completed the process evaluation questionnaire (i.e., satisfaction with the program) used in the original RCT (21).

#### 2.4.3 Physical fitness test and resistance training skills battery

The teachers were provided with the test instructions and printable test sheets. They were informed to conduct the tests before and after the 8-week program. Each teacher organized the test on their own and involved the students to record the results and uphold the quality requirements. The physical fitness tests included the standing long jump, push-up, and beep tests. The purpose of a standing long jump test was to measure the students' lower body power and was used because it was a valid measurement of youth (33). The student started the test by standing behind a line and then did their longest jump. The distance was measured from the start line and to the heels of the nearest foot. Students completed the jump twice and the longest jump in centimeters was used for the evaluation. The 90°push-up-test was used to evaluate students' upper body muscular endurance. This is also a valid measurement of youth (30). The test started with the students standing in plank position and a classmate counting the repetitions. Each repetition was counted in the measurement when the student could go down to a 90° angle at the elbows, could keep the body straight, and did it to the rhythm of 40 bpm cadence. Finally, the students were tested on their aerobic capacity using the beep-test, also called the 20-m shuttle run test (34). In the original RCT study, the students completed a step-up test to measure their VO2 max with a heart rate monitor (21). Due to practical reasons (i.e., availability of step platforms) and due to the integration of the beep-test in the RT4T smartphone app the beep-test was used to assess their aerobic capacity. The test started by standing behind a line and then running to the line 20 m apart prior to the sound of a beep. Then they had to cross the opposite line before a beep in an increasing pace. The students' results were the final completed shuttle. The evaluation was in meters and the 0.1 level is 20 m.

The Resistance Training Skills Battery was used to assess students' progress, provide feedback, and measure movement skill competency (35). It consist of six exercise with four or five defined performance criteria, capturing essential movement proficiency relevant to resistance training in adolescents (35).

#### 2.4.4 Interviews

The purpose of the interviews was to elaborate on the quantitative data and to gain knowledge of the experiences with RT4T (explanatory sequential mixed methods design). The interviews where structured as group interviews with 4–5 students or 2 teachers and followed a semi-structured guide, which was based on key elements of evaluating feasibility studies: Acceptability, fidelity, reach, dosage, adaption, resources, motivation, and maintenance (24, 26, 36, 37). The interviews were led by the first author and lasted between 20 and 45 min.

### 2.5 Analyses

#### 2.5.1 Quantitative analyses

Paired sample *t*-tests were used to compare students' performance on the physical fitness tests and surveys before and after the RT4T program using Stata BE 17. A paired sample *t*-test was used due to the repeated measurements in the same small group of participating students.

#### 2.5.2 Qualitative analyses

The group interviews were fully transcribed, and a deductive content analysis approach was used lead by the first author (38). The deductive content analysis is a structured strategy that allows the use of predetermined codes from literature or theories to approach the data. The analysis followed three phases: Preparation, Organization, and Reporting. The first phase was to get familiar with and prepare the data by reading all the transcribed interviews a couple of times. The next step was to organize the data deductively using codes from implementation literature in unconstrained analyses matrix: Acceptability, Fidelity and Adaptations, and Delivery and Support (24–26, 36). Within each theme the data was grouped, categorized, interpreted and reported (38).

### 2.6 Ethics approval and consent

This feasibility study was approved by the Research Ethics Committee of South Danish University in August 2022 (ID 22/24049) and the data processing was accepted by the SDU Research and Innovation Organization (ID 11.606). This was a condition for the collaboration with the University and Newcastle in Australia. Both teachers, students, and parents were provided with informed consent to provide the results from measurements prior to and after the 8 weeks of RT4T. Moreover, all involved were provided with information about RT4T and data processing.

## 3 Results

### 3.1 Participant flow and baseline characteristics

A total of six classes from two schools participated in the feasibility trial. All students participated in the RT4T sessions during PE and written informed consent to be included in the research study was obtained from 67 students. A total of 57 participants answered both rounds of survey and were included in the study. The mean age was 13.7 years, and a small majority were from the 7th-grade classes (56.1%) and males (68.0%) (Table 2). Average number of self-reported days with moderate to vigorous physical activity for at least 60 min was 4.3 days at baseline (Table 3).

**Table 2:** Characteristics of the included participants.

Characteristics	RT4T intervention (*n* = 57)
Age, mean (SD)	13.7 (1.13)
Male, participants *n* (%)	39 (68.4%)
7th-grade classes (%)	32 (56.1%)
9th-grade classes (%)	25 (43.9%)

**Table 3:** Results of the physical fitness test, RT skills battery and survey.

	*N* ^a^	Baseline (95% CI)	Follow-up (95% CI)	Mean difference	*t*-value	*p*-value
Physical tests						
Push-ups (repetitions)	50	16.6 (14.5–18.7)	17.7 (14.46–20.9)	1.1	0.9	0.351
Standing long jump (cm)	49	172.8 (163.4–182.3)	176.9 (167.46–186.4)	4.1	1.3	0.194
Beep-test (m)	50	712.0 (599.1–824.9)	914.4 (754.8–1,074.0)	202.4	3.2	0.002
RT Skills Battery						
Squat^b^	18	4.3 (3.8–4.9)	4.5 (4.2–4.9)	0.2	0.7	0.483
Push-ups^c^	18	3.7 (3.1–4.2)	4.1 (3.8–4.4)	0.4	1.6	0.134
Lunges^b^	18	4.3 (3.6–4.9)	4.7 (4.5–5.0)	0.4	1.4	0.177
Overhead press^b^	15	3.6 (3.1–4.2)	3.6 (3.2–4.0)	0.0	0.0	1.000
Front support chest touches^b^	18	4.2 (3.7–4.8)	4.4 (4.1–4.7)	0.2	0.7	0.483
Suspended row^c^	15	3.3 (2.7–4.0)	3.6 (3.0–4.2)	0.3	0.6	0.556
Perceived fitness						
General fitness^d^	56	3.4 (3.2–3.7)	3.5 (3.3–3.7)	0.1	0.5	0.626
Cardiorespiratory fitness^d^	56	3.2 (2.9–3.4)	3.3 (3.1–3.6)	0.2	1.4	0.161
Muscular fitness^d^	56	3.2 (3.0–3.5)	3.4 (3.2–3.7)	0.2	1.9	0.057
Speed/agility^d^	56	3.5 (3.2–3.7)	3.6 (3.3–3.8)	0.1	1.0	0.321
Flexibility^d^	56	2.7 (2.4–3.0)	2.7 (2.5–3.0)	0.1	0.6	0.568
Self-reported physical activity						
Physical activity (days/week)	56	4.3 (3.8–4.8)	4.5 (4.1–4.9)	0.2	0.9	0.355
Exercise autonomous motivation						
Identified regulation^e^	57	3.5 (3.2–3.8)	3.5 (3.2–3.7)	0.0	0.2	0.869
Intrinsic regulation^e^	57	3.7 (3.4–3.9)	3.6 (3.4–3.9)	0.0	0.3	0.738
Well-being and self-efficacy						
RT self-efficacy^f^	56	3.8 (3.6–3.9)	4.0 (3.8–4.2)	0.2	2.9	0.005
Well-being^g^	57	45.9 (44.3–47.5)	43.6 (41.1–46.2)	−2.2	−1.7	0.097

^a^ Participants with baseline and follow-up data.

^b^ Fulfilled criteria out of five.

^c^ Fulfilled criteria out of four.

^d^ 5-point scale (poor to excellent).

^e^ Average of four questions on a 5-point scale (not true for me to very true for me).

^f^ Average of four questions on a 5-point scale (strongly disagree to strongly agree).

^g^ Sum of eight questions on a 7-point scale (strongly disagree to strongly agree).

### 3.2 Quantitative analyses

The outcome evaluation is presented in Table 3. We observed small non-significant increases in the push-ups and standing long jump test results. Running distance on the beep test increased from 712.0 m to 914.4 m (*p *= 0.002). Only one school completed both rounds of the Resistance Training Skills Battery due to lack of time. For the students who did both rounds, we found small non-significant increases for most items. Small insignificant increases were also found for self-reported physical activity and perceived fitness, with muscular fitness approaching significance (*p *= 0.057). Motivation for physical activity (BREQ-2) was almost unchanged, while there were found a significant increase in Resistance Training Self-Efficacy (*p *= 0.005).

Table 4 presents the mean results of four questions related to the students' satisfaction with the program. The students' overall rating of the program was just above average at the 1–5 scale with a mean of 3.1, while the rating of teachers' delivery was 3.2. Average of the enjoyment of the sessions was 3.3 on the 1–5 scale, and most of the students never used the smartphone app due to technical and practical issues (1.3).

**Table 4:** Students’ satisfaction with the 8-week RT4T program (*n* = 56).

Question	Mean (SD)
Rating of program^a^	3.1 (1.1)
Rating of teacher delivery^a^	3.2 (1.0)
Session enjoyment^b^	3.3 (1.1)
Usage of smartphone app^c^	1.3 (0.7)

^a^ 5-point scale (poor to excellent).

^b^ 5-point scale (strongly disagree to strongly agree).

^c^ 4-point scale (never to often).

### 3.3 Qualitative analyses

The analyses of the interviews with teachers and students are presented in Table 5 in the following three themes: (1) Acceptability and Relevance, (2) Fidelity and Adaptation, (3) Delivery and Support.

**Table 5:** Qualitative analyses of two interviews with teachers and two group interviews with students (school 1: 9th grade; school 2: 7th grade).

Central themes	Key points	Examples
Acceptability	– Teachers found it relevant and were inspired by the activities and the structure of the sessions	✓ *… this is one of the areas we must work with according to Common Goals is Physical Education, so I think this could be a good structure to build the area up around…* (Teacher 1, School 2)
	– Students were familiar with the exercises, and they were able to participate with different competencies	✓ *… There were exercises I had not tried before, but it was not because I did not know them…* (Student 3, School 1). ✓ *So, they (exercises) got scaled to those (Students) who was not that good* (Student 3, School 2)
	– Students liked the new activities and equipment, but motivation declined during the 8-weeks.	✓ *… 5 weeks, where we have done the same at every session (…) in the end it got very boring…* (Student 4, School 1) ✓ *…when it is just resistance training for so long, they lost the concentration a little* (Teacher 2, School 1)
Fidelity and adaptations	– Structure, activities, and materials were used as intended, but different parts were prioritized or left out	✓ *We had a good structure (..) the sessions were built up in the same way each time …, [but] I really often forgot the last part with stretch and cool down…* (Teacher 1, School 2) ✓ *The boxing and music challenges were the part… where they forgot themselves and just were committed* (Teacher 1, School 1)
	– Providing choices for the students were not prioritized	✓ *I did not give the students any options* (Teacher 1, School 2)
	– Some teachers experienced that the students disregarded the test criteria during the physical fitness tests, which questions the reliability of the test results	✓ *… it was difficult to score correct because they cheated…. in one way or another, they mostly helped each other with the technique during testing. (Teacher 2, School 1)*
Delivery and support	– Some teachers found it difficult to adjust the activities to different skills levels, and to provide students with useable feedback	✓ *All could participate, but it was difficult to challenge everybody unless you really had to differentiate…* (*T*eacher 2, School 1)
	– The equipment and circuit cards were motivating and useful, but the Gymstics were difficult to use for some.	✓ *… the students take up the new exercises without coaching them during the session (…) and then I could focus on the students, who needed it… (Teacher 2, school 2)* ✓ *It was more fun with the boxing gloves, because the other exercises were exercises you could do at home… (Student 1, School 2)* ✓ *I liked the physical objects… (Teacher 2, school 1)*
	– The smartphone app was not used due to technical and practical issues, and the students had difficulties to understand some of the information on the circuit cards in English.	✓ *It was a huge challenge to log in* (Teacher 2, School 2) ✓ *… It is difficult to interpret the description when it is on English…* (Teacher 1, School 1)

Teachers and students found the activities and structure of the sessions relevant and inspiring. The students were familiar with many exercises and could participate at different competency levels. Although students initially enjoyed the new activities and equipment, their motivation decreased over the 8-week period.

The implementation of the program demonstrated fidelity with some adaptations: the intended structure, activities, and materials were used, but certain parts were prioritized, and others omitted. For instance, the cool-down part was sometimes forgotten, and the HIRT sessions were shortened to allocate more time to other activities, driven by student motivation. However, providing choices for students was not prioritized. Additionally, during physical fitness test recordings, some teachers observed overly friendly behavior among peers, raising concerns about the reliability of the test results due to potential cheating and excessive assistance with technique.

Teachers faced challenges in including students with different skill levels and providing effective feedback. While all students could participate, differentiation was a struggle for some teachers. The equipment and circuit cards were motivating and useful, but the Gymsticks™ (elastic resistance bands) posed difficulties for some. Boxing gloves were particularly enjoyable for most students. The smartphone app wasn't utilized due to technical and practical issues, and English language barriers caused difficulties for students in understanding information on the circuit cards.

## 4 Discussion

The aim of our study was to investigate if the RT4T program could be adapted and feasibly delivered in two Danish schools and produce similar effects to those observed in the original study. More specifically, our study was designed to determine if teachers are willing and able to deliver the RT4T program—and if it works as effectively in the Danish context?

### 4.1 Are teachers willing and able to deliver the RT4T program?

Recruitment of teachers and schools to the trial was difficult and before scaling up RT4T in Denmark attitudes, possibilities and recruitment strategies should be investigated further. This could be due to general lack of time, project overload, as well as reluctance to deliver resistance training in PE at the cost of other activities.

Participating teachers were very motivated for the program and found it very relevant. They found it aligned with elements of the PE curriculum under the mandatory theme “Physical Training”. They were able to deliver the sessions and received average ratings from the students' satisfaction questionnaire. These satisfaction ratings in Denmark were lower than the ratings observed in the original study (21). The teachers tried to build a supportive climate and verbally motivated students during the activities. One of the teachers focused on skill acquisition and asked the students to show or try new skills. All teachers tried to raise the activity levels but expressed the difficulties in supporting all students. Furthermore, the teacher expressed limitations in their experience with the new exercises and difficulties with providing the appropriate feedback and support to all students. One teacher found it difficult to prioritize the cool-down because of a lack of time, and another teacher used less time on the HIRT and more time on the BoxFit and GameFit to increase the motivation and enjoyment.

The primary aim of the RT4T program is to provide adolescents with competence, confidence, knowledge, and motivation to participate in resistance training. In the interviews the students expressed confidence with doing the exercises and the ability to adapt the activities to their own level. They were familiar with most of the exercises, but most of the students were not experienced with all of them before the intervention. One of the pillars in the SAAFE principles is to build autonomous motivation but providing students with choice. One teacher did not provide any choice at all, while another teacher only provided choice during parts of the program. The students expressed a lack of choice, and they recommended it as *a priori*ty for future trials and scale-up. Finally, students' motivation decreased during the 8-week program, and some of the students found it to be too long as they are accustomed to more variation in PE. Both students and teachers suggested more variation in future trials of the program. The variation could consist of different locations, training methods, and/or equipment.

Adaptations and flexibility are necessary in school-based programs, but the risk of missing out on key components and mechanisms are present (39). Some adaptations could be grounded in misconceptions of the intervention mechanisms and key components, or it could be due to an insufficient training and support system unable to develop the necessary teaching competences and quality in delivery. Another explanation could be grounded in cultural differences in PE practices and curricular requirements (36). The Danish teacher might be too flexible in their approach and adapt too much, which previously have led to a call for prioritization of description, promotion and evaluation of quality and wanted adaptations in school-based interventions (40).

### 4.2 Did the program work as well as the original?

In the beep-test the 50 students had a significant increase on 202.4 m. This increase is likely to be caused by other factors than improvements in aerobic capacity e.g., different motivations at the two test rounds or issues related to the field test set-up and the involvement of students as test personnel (41–43). It is not possible to compare the results directly with the original study, because they used the step-up test, but they found no significant effects (21). Other school-based resistance training interventions have found improvements in aerobic capacity, with two sessions per week (9).

For upper body strength, 50 students participated in both rounds of push-ups test. These results are directly comparable with the original studies, where a significant intervention effect from 12.3 to 14.1 push-ups were found after 6-months in the RCT study (21) and an increase from 15.2 to 18.4 in the scale-up study after 10-weeks (20). We found a smaller insignificant increase from 16.6 to 17.7 push-ups. Our average increase of one repetition may be attributable to the smaller number of sessions completed by participants in the current study (i.e., one session per week compared with two sessions/week in the previous cluster RCT). Previous resistance training studies with either one or two sessions a week found an intervention effect in the push-ups test after a school-based intervention (9, 14–16).

For the standing long jump test, the 49 students increased their jumping distance within average 4.1 cm (*p* = 0.194). In the original RCT study the intervention group increased their jumping distance from 164.4 cm to 166.6 cm after 6 months (21), and from 170.6 cm to 176.0 cm in the scale-up study (20). The increase in the Danish study is therefore approximately comparable with the original effects and a larger study population might have resulted in statistically significant effects. Previous school-based resistance training interventions have found intervention effects on the standing long jump with two sessions per week (9, 14, 16).

Overall, based by the evidence from the current feasibility trial lower body power reached approximately the same effect sizes as the original studies, upper body muscular endurance a little less and aerobic capacity in not comparable. The test set-up was organized without researcher and experts and relied solely on the teachers and the students themselves, who recorded rounds, lengths, and repetitions. The teachers observed that some of the students disregarded quality standards when judging each other in the push-ups test and standing long jump test. This contributed to a decrease in the reliability of the test results. On the other hand, it is important for the students' experience and learning, and it decreases the cost of the intervention and make it easier to potentially implement the interventions at scale at a later timepoint once the stage of initial testing is mastered.

As mentioned earlier, the aim of the RT4T program is to promote competence, confidence, knowledge, and motivation, and therefore the measured outcome of students' own perceptions of fitness, self-efficacy and motivation is important. In the current feasibility, we observed a significant increase in resistance training self-efficacy consistent with the original RCT and scale-up studies. Furthermore, we also observed small increases in perceived fitness from 0.1 to 0.2 points. This is also in line with the Australian scale-up study, which found increases of ∼0.2-points. For the autonomous motivation for exercise and for general well-being there were found no difference or a small decline. This is also the case for the original studies.

### 4.3 Implication for progression

As stated in the CONSORT guidelines, the rationale of pilot and feasibility studies is to investigate areas of uncertainty about a future definitive RCT (24). Or in a case like the current trial, to adapt and test an evidence-based program conducted in one setting to another. One of the points in the CONSORT checklist is to interpret the results of the study and make the implication for progression towards further upscaling. Based on the results presented three points for amendments and improvements will be underlined.

• It was difficult to recruit teacher and schools to the study, and before progression to further trials or scale-ups it is important to investigate root causes to this and possible solutions and recruiting strategies.
• Training and ongoing support/supervision could be improved, and could be informed by the latest research on effectiveness of different implementation support strategies from the Australian research group (44) and others (45). Teachers and students experienced low adherence to the SAAFE principles, and teacher experienced difficulties giving feedback and ensuring quality in the exercises for all students. Furthermore, the circuit cards should be translated to Danish to improve the students understanding of the exercises and written instructions. Finally, the smartphone app was largely not used in this trial due to practical and technical issues. Adaption and test of the smartphone app or alternative solutions is necessary before proceeding.
• The motivation was decreasing during the 8-week period, and the students did not experience choices in most of the sessions. Future trials should investigate how students could maintain motivation through e.g., increased variation, progression, and choice during PE in 7th to 9th grade.

### 4.4 Strengths and limitations

This study adapted and evaluated an evidence-based program across countries and cultures. The adaptations of materials and resources were guided by the PRACTIS guidelines and included guidance from the research team from University of Newcastle as well as insights from the participating teachers. The tests have included two participating schools with two different age groups, and have used a mixed methods approach including surveys, physical fitness tests, and interviews with teachers and students. The different methods complement each other and target different parts of the aim. The questionnaire underwent a rigorous translation and comparison process and the included scales showed high internal consistency with Cronbach's alpha values over 0.8. However, we are not aware of any studies that have conducted additional psychometric testing (e.g., test-retest reliability) of the Danish versions of the scales. Only two teachers participated in the adaptation process, and the adapted program was only tested on two schools. More teachers are needed to test the program and materials before progressing to a definitive trial. Despite the possibility to compare the effects with the original studies and short follow-up period a control group would have improved the study design due to potential maturation effects and learning effects related to the physical tests. Another limitation is the relative low share of students' and overweight of boys consenting to participate in the study and providing test and survey data. Even though all students participated in the program during PE, the low participation rate and small gender imbalance in the study make us less certain on the results. One school did not finalize the second round of Resistance Training Skills Battery, and therefore it was not possible to use these tests. Finally, the physical fitness test set-up and preparation should be further improved to ensure reliability of the outcome measures.

## 5 Conclusion

The current study translated and adapted an evidence-based resistance training program to Danish school context and found it to be acceptable and feasible. The teachers found the program to be relevant and were helped by the structure, activities, equipment, and principles in the program. Students were able to participate in the program, and were motivated by the new activities, the equipment, and the game-based activities. Furthermore, students were motivated by the physical fitness test and to track their progress. The preliminary efficacy evaluation showed positive developments comparable to the original studies in most measures and tests.

Before progressing to further upscale of the RT4T-program in Denmark further studies and program refinements are needed. Firstly, the interest of resistance training programs should be investigated among a larger population of teachers and schools. Secondly, the training of the teachers should be improved by providing guidance and feedback during the 8-week period, the smartphone app should be made available and circuit cards should be translated to Danish. Finally, the finding that motivation was declining during the 8-week should be considered and different modes of delivery and structure should be tested further.

## Data Availability

The raw data supporting the conclusions of this article will be made available by the authors, without undue reservation.
